# Intereye Microvascular Differences in Patients With Same-Stage Diabetic Retinopathy Revealed by OCTA

**DOI:** 10.1167/iovs.65.6.11

**Published:** 2024-06-06

**Authors:** Heiko Stino, Kim Lien Huber, Laura Kunze, Irene Steiner, Alexandros Bampoulidis, Ursula Schmidt-Erfurth, Andreas Pollreisz

**Affiliations:** 1 Medical University of Vienna, Department of Ophthalmology, Vienna, Austria; 2 Medical University of Vienna, Center for Medical Data Science, Institute of Medical Statistics, Vienna, Austria; 3 Medical University of Vienna, Vienna Reading Center, Department of Ophthalmology, Vienna, Austria

**Keywords:** diabetic retinopathy, OCTA, microvasculature, intereye differences, retinal blood flow

## Abstract

**Purpose:**

To evaluate microvascular intereye differences in diabetic patients with same-stage diabetic retinopathy (DR) in both eyes as assessed using optical coherence tomography angiography (OCTA).

**Methods:**

In this cross-sectional study, fovea-centered swept-source 6 × 6 mm OCTA scans were acquired using a 200 kHz OCTA device. Vessel density (VD) and fractal dimension were calculated on binarized, vessel-segmented images in the superficial capillary plexus (SCP) and deep capillary plexus (DCP). Foveal avascular zone (FAZ) area (FAZA) and perimeter (FAZP) was measured and FAZ circularity (FAZC) calculated. Absolute difference (δ_abs_) and asymmetry index between eyes was assessed and compared across DR stages. Differences of VD, FD, and FAZ parameters between left and right eye were evaluated using linear mixed models.

**Results:**

A total of 336 eyes of 168 diabetic patients without DR and with DR stages ranging from mild nonproliferative to proliferative DR were included for analysis. The intereye comparison revealed significantly lower VD in the SCP (estimate [95% CI] = −0.009 [−0.01; −0.006], *P* < 0.01), as well as a significantly lower FD in the SCP (−0.007 [−0.009; −0.005], *P* < 0.01) of the left compared to the right eye. FAZC of the left compared to the right eye was lower in eyes without DR, moderate DR, and PDR (*P* < 0.05). FAZ δ_abs_ and asymmetry index were higher in more advanced disease stages (*P* < 0.05).

**Conclusions:**

OCTA metrics provide important information on the retinal microvasculature in systemic diseases such as DR. Our results reveal a significant intereye difference with lower VD and FD in the SCP as well as higher FAZ impairment of the left compared to the right eye.

In the next two decades, the number of patients with diabetes is expected to increase to 700 million people worldwide of which about 20% to 35% will suffer from any form of diabetic retinopathy (DR).^1^^–^^3^ Because DR develops and progresses asymptomatically in early stages, screening programs are substantial to diagnose and treat patients before irreversible damages occur.^4^

Although DR stage is traditionally assessed by detecting diabetic lesions on color fundus (CF) images as reported by the Early Treatment Diabetic Retinopathy Study (ETDRS), the development of novel devices have provided new ways to analyze changes to the microvasculature.^5^ Capillary dropout caused by the ongoing vasculopathy in DR can be investigated by analyzing retinal vessel density (VD) on optical coherence tomography angiography (OCTA) en-face images. Microvascular changes seem to precede clinically detectable lesions during fundus examination because decreased VD could be observed in diabetic patients without DR.^6^ Another potentially valuable method for assessment of microvascular structure is the analysis of fractal dimension (FD) as a global measure for vascular complexity expressed as a single variable. Zahid et al.^7^ found FD assessed on OCTA to be significantly reduced in the SCP and DCP of eyes with DR compared to healthy controls.

In patients with DR, various authors have evaluated those retinal parameters describing an association of reduced VD in the superficial capillary plexus (SCP) with diabetic macular edema (DME) development and lower retinal sensitivity and a reduction of VD and FD in the deep capillary plexus (DCP) with DR progression and visual acuity (VA).^8^^–^^11^

Measurements of the foveal avascular zone (FAZ) were reported in numerous publications. Enlargement of the FAZ area (FAZA) was shown to be negatively correlated with VA and positively with DR severity and progression.^9^^,^^12^^,^^13^ FAZ circularity (FAZC) was established as a more robust factor for microvascular analysis showing a significant reduction as DR progresses and as FAZ becomes more irregular.^14^^–^^16^ Investigation of subclinical parameters seems essential because DR affects the microvasculature before clinical signs present.^6^

Although a systemic disease, diabetes seems to affect eyes of the same patient at a different degree.^17^ Although a recent study investigated asymmetry only between eyes, we hypothesize that these differences are not randomly distributed but caused by asymmetrical blood flow of the left and right carotid artery through hemodynamic stress.^18^^,^^19^ This study aims to evaluate and compare OCTA-based microvascular intereye differences between the left and right eye in diabetic patients with the same DR stage in both eyes.

## Methods

This cross-sectional study was conducted at the Department of Ophthalmology, Medical University of Vienna. Approval was obtained from the ethics committee of the Medical University of Vienna (1858/2018). The study adhered to the Declaration of Helsinki. All patients provided written informed consent. Diabetic patients were recruited from the outpatient clinic of the unit for diabetic ocular diseases after routine clinical examination including visual acuity, slit-lamp examination, and OCT (Heidelberg Spectralis; Heidelberg Engineering, Heidelberg, Germany). DR stage was clinically assessed according to the International Clinical Diabetic Retinopathy Severity Scale on color fundus images (Clarus 700; Carl Zeiss Meditec Inc., Dublin, CA, USA).^20^ Patients with myopia ≤ −3 and hyperopia of ≥ 3 diopter, history of previous retinal disease, trauma, severe media opacity, and presence of DME on OCT scans were excluded from further analysis.

After pupil dilation using mydriatic eye drops (0.5% Tropicamide), fovea-centered 6 × 6 mm OCTA scans were acquired using a swept-source Zeiss PLEX Elite 9000 swept source OCTA at an A-scan rate of 200kHz, 1060 nm central wavelength, 20 µm lateral resolution, 6µm axial resolution, and a scanning depth of 6 µm (Carl Zeiss Meditec Inc., Dublin, CA, USA). The sequence in which both eyes were acquired was randomized using a validated automated randomization tool (https://www.randomizer.at/). Because previous studies reported a high repeatability for OCTA-based measurements, only one image per eye was acquired.^21^^–^^23^ Acquisition was repeated if a reduced image quality was noticed by the operator. Automated segmented SCP and DCP en-face images were exported for analysis of VD and FD. Image quality was assessed by two independent graders (KLH, LK) and, in cases of severely reduced quality, excluded by a supervising grader (HS).

VD was calculated as the percentage of vessel area with blood flow over the total area measured. To evaluate the microcirculation of the macula, the larger vessels were excluded from the calculation of the VD in the SCP. If present, projection artefacts in the DCP were excluded from analysis. VD analysis was performed using an in-house software developed by the Vienna Reading Center as previously reported.^24^ The software Fractalyse was used to calculate FD with the box-counting method, as most commonly reported in the literature.^25^^,^^26^

**Figure 1.**
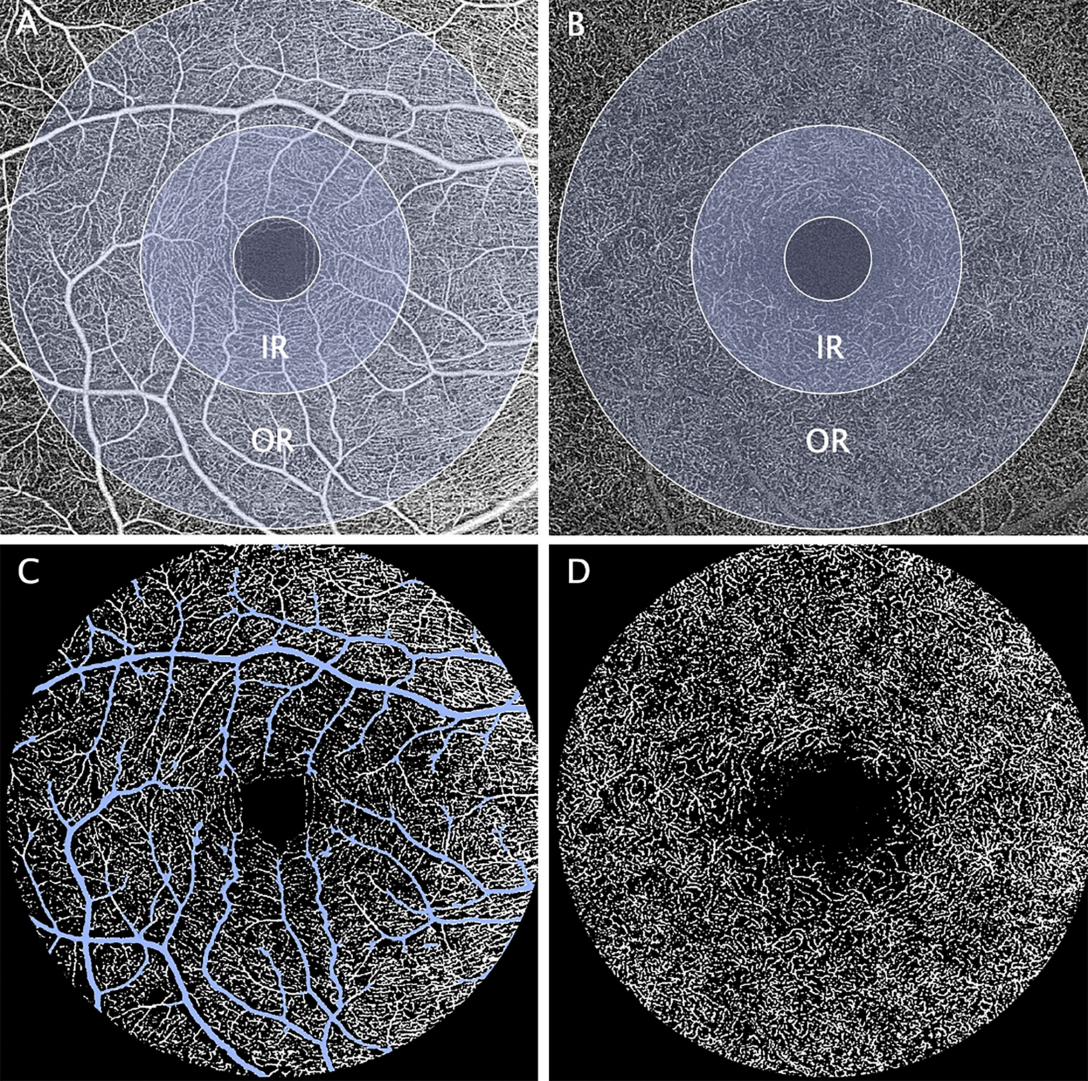
Fovea-centered 6 × 6 mm OCTA en-face image of the SCP and DCP in a patient with moderate diabetic retinopathy. Automated segmented SCP (**A**) and DCP (**B**) are shown with the overlaid ETDRS grid consisting of an IR and an OR. Binarized images of the SCP (**C**) and DCP (**D**) are used for analysis of VDs and FDs after exclusion of the big vessels (highlighted in *blue*).

Both calculations were performed on binarized, vessel-segmented images that were generated by applying thresholding. The threshold of each image is defined as:

threshold=std_coeff*sd+mean

where mean and sd are the mean and standard deviation of the pixel values of the image, and std_coeff set to 0.5 when segmenting all vessels and 1.3 when segmenting the big vessels only (Fig. 1). The thresholds were chosen empirically by the Vienna Reading Center.

VD was calculated in all individual sectors (inner ring [IR]; outer ring [OR]) and in the total area of the 6 mm ETDRS grid centered around the fovea. To place the grid, the center of the FAZ was manually annotated on the en-face OCTA image using ImageJ (National Institutes of Health, Bethesda, MD, USA).

**Figure 2.**
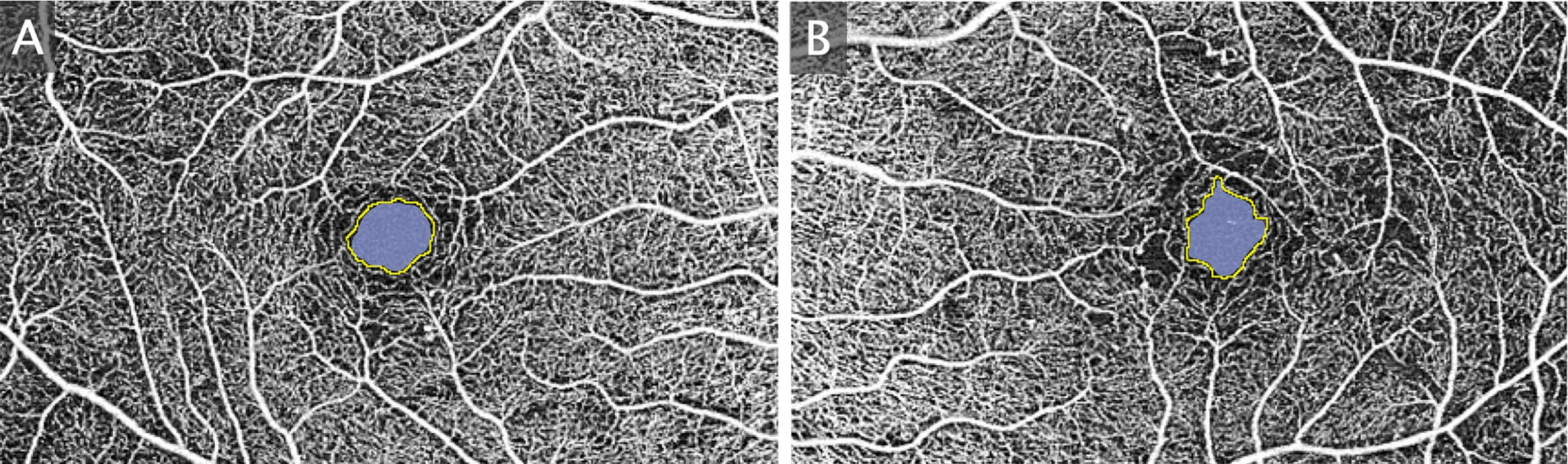
Fovea-centered 6 × 6 mm OCTA en-face image of the superficial capillary plexus with FAZ measurements of the right (**A**) and left eye (**B**) of the same patient. FAZ perimeter (*yellow line*) and area (*blue area*) are measured manually.

FAZA and FAZP were measured on en-face images of the SCP using ImageJ (National Institutes of Health) (Fig. 2). FAZC was calculated from FAZA and FAZP using the formula reported in the literature^14^^,^^27^^,^^28^:

$$\text{Circularity}=4*\pi \;\frac{Area}{Perimeter^{2}}$$

Absolute differences (δ_abs_) between eyes and asymmetry index as recently reported by Zhao et al.^18^ was calculated for each patient as the absolute difference between eyes divided by the mean of the two eyes, multiplied by 100.

### Statistical Analysis

Exploratory data analyses were performed. For quantitative variables, mean ± standard deviation are reported if the variable is approximately normally distributed, and median (first quartile [Q1]; third quartile [Q3]) otherwise. For comparison of DR stages between asymmetry index or absolute difference with regard to VD, fractal dimension (in the deep and superficial plexus), and FAZ parameters, Kruskal-Wallis-tests were calculated. Comparisons of asymmetry index or absolute difference in VD between inner and outer ring was done by Wilcoxon signed-rank tests. To analyze the association of side and stage with VD, fractal dimension and FAZ parameters, linear mixed models were calculated. The independent variables were DR stage, side (right vs. left), age, HbA1c, sex, and the interaction between stage and side. Patient was taken as a random factor. Derived from these mixed models, F-tests were calculated for DR-stage. If the *P* value of the *F* test was < 0.05, comparisons to the category “no DR” were calculated. No multiplicity correction was performed. Hence, the interpretation of the *P* values is descriptive. Significance level was set to α = 0.05. Statistical analyses were conducted with R 4.2.1.^29^

## Results

OCTA scans of 352 eyes of 179 diabetic patients were acquired. Ten images of five patients (3%) were excluded after quality assessment because of severely reduced image quality. In six patients imaging was only possible in one eye, respectively, as reduced compliance resulted in motion artifacts, also leading to the exclusion of those six eyes.

For final analysis 336 eyes of 168 patients with a mean age of 57.2 ± 13.1 years (111 male, 57 female) were included. Mean HbA1c was 7.34 ± 1.32 in all patients (n = 167). For one patient HbA1c was unknown. Twenty-five percent (n = 42) of patients had type 1 and 75% (n = 126) type 2 diabetes. According to the International Clinical Diabetic Retinopathy grading no, mild, moderate, severe nonproliferative DR (NPDR), and PDR was assessed on CF images in both eyes of 89 (53%), 19 (11.3%), 26 (15.5%), 14 (8.3%), and 20 (11.9%) patients, respectively. Quiescent PDR was assessed in five eyes graded as PDR.

In the entire sample, mean VD of the right versus left eye in the SCP and DCP was 0.218 ± 0.018 versus 0.209 ± 0.019 and 0.233 ± 0.019 versus 0.223 ± 0.021; with a mean delta of 0.016 ± 0.012 and 0.011 ± 0.01, respectively. Mean FD of the right versus left eye in the SCP and DCP was 1.770 ± 0.014 versus 1.763 ± 0.015 and 1.776 ± 0.013 versus 1.775 ± 0.014; with a mean delta of 0.012 ± 0.01 and 0.008 ± 0.007, respectively.

Mean FAZA of the right versus left eye was 0.258 ± 0.185 mm^2^ versus 0.254 ± 0.151 mm^2^; with a mean delta of 0.043 ± 0.063 mm^2^. Mean FAZP of the right versus left eye was 2.027 ± 0.702 mm versus 2.084 ± 0.685 mm; with a mean delta of 0.211 ± 0.228 mm. Mean FAZC of the right versus left eye was 0.747 ± 0.091 versus 0.706 ± 0.106; with a mean delta of 0.067 ± 0.058. Detailed values of VD and FD of the SCP and DCP, and FAZ parameters in different stages of DR are specified in Table 1.

**Table 1.** VD, FD, and FAZP in Different Stages of DR

Eye	No NPDR	Mild NPDR	Moderate NPDR	Severe NPDR	PDR
VD SCP					
Right	0.218 ± 0.017	0.227 ± 0.017	0.218 ± 0.019	0.213 ± 0.024	0.212 ± 0.018
Left	0.209 ± 0.019	0.217 ± 0.018	0.207 ± 0.021	0.203 ± 0.023	0.21 ± 0.016
VD DCP					
Right	0.239 ± 0.015	0.238 ± 0.015	0.232 ± 0.016	0.222 ± 0.028	0.215 ± 0.028
Left	0.239 ± 0.017	0.238 ± 0.014	0.231 ± 0.014	0.219 ± 0.033	0.216 ± 0.023
VD SCP IR					
Right	0.215 ± 0.035	0.223 ± 0.038	0.22 ± 0.043	0.209 ± 0.035	0.195 ± 0.033
Left	0.207 ± 0.036	0.211 ± 0.04	0.2 ± 0.038	0.191 ± 0.04	0.194 ± 0.034
VD SCP OR					
Right	0.302 ± 0.019	0.309 ± 0.018	0.3 ± 0.019	0.299 ± 0.033	0.302 ± 0.024
Left	0.291 ± 0.019	0.299 ± 0.014	0.289 ± 0.022	0.289 ± 0.023	0.296 ± 0.02
VD DCP IR					
Right	0.259 ± 0.037	0.267 ± 0.031	0.26 ± 0.033	0.26 ± 0.032	0.256 ± 0.044
Left	0.257 ± 0.036	0.264 ± 0.026	0.25 ± 0.044	0.25 ± 0.038	0.264 ± 0.037
VD DCP OR					
Right	0.278 ± 0.015	0.275 ± 0.018	0.271 ± 0.016	0.265 ± 0.025	0.262 ± 0.017
Left	0.277 ± 0.017	0.275 ± 0.014	0.271 ± 0.018	0.262 ± 0.034	0.263 ± 0.017
FD SCP					
Right	1.771 ± 0.013	1.775 ± 0.012	1.770 ± 0.013	1.764 ± 0.021	1.759 ± 0.015
Left	1.764 ± 0.014	1.768 ± 0.014	1.762 ± 0.015	1.757 ± 0.019	1.756 ± 0.011
FD DCP					
Right	1.78 ± 0.01	1.778 ± 0.01	1.776 ± 0.01	1.767 ± 0.02	1.762 ± 0.013
Left	1.78 ± 0.012	1.777 ± 0.009	1.774 ± 0.01	1.764 ± 0.024	1.764 ± 0.013
FAZA					
Right	0.218 ± 0.088	0.259 ± 0.121	0.256 ± 0.116	0.34 ± 0.147	0.379 ± 0.435
Left	0.217 ± 0.091	0.243 ± 0.093	0.243 ± 0.113	0.342 ± 0.114	0.385 ± 0.304
FAZP					
Right	1.839 ± 0.37	2.049 ± 0.604	2.032 ± 0.521	2.42 ± 0.611	2.557 ± 1.472
Left	1.871 ± 0.393	2.029 ± 0.51	2.04 ± 0.48	2.465 ± 0.569	2.871 ± 1.289
FAZC					
Right	0.774 ± 0.072	0.757 ± 0.101	0.746 ± 0.085	0.713 ± 0.073	0.643 ± 0.105
Left	0.743 ± 0.08	0.723 ± 0.107	0.696 ± 0.089	0.69 ± 0.076	0.55 ± 0.107

### Intereye Difference

Absolute intereye differences (δ_abs_), as well as asymmetry indexes of VD, FD, and FAZ parameters in different stages of DR and ETDRS rings, are specified in Table 2. Kruskal-Wallis tests did not reveal any significant differences in VD and FD of asymmetry index or absolute intereye differences between DR stages (*P* values ranging from 0.06 to 1). However, we observed higher values in IR compared to OR in both, the SCP and DCP. This observation was confirmed by Wilcoxon signed rank tests (SCP: asymmetry index: median [Q1; Q3]: 6.69 [1.78; 14.4], *P* < 0.0001, δ_abs_: 0.009 [−0.002; 0.026], *P* < 0.0001; DCP: asymmetry index: 4.42 [−0.27; 9.73], *P* < 0.0001, δ_abs_: 0.010 [−0.0002; 0.025], *P* < 0.0001).

**Table 2.** Median (First Quartile; Third Quartile) of Asymmetry Index and Absolute Difference Between Right and Left Eye in Different Stages of DR

	No NPDR	Mild NPDR	Moderate NPDR	Severe NPDR	PDR
VD SCP					
δ_abs_	0.013 (0.008; 0.025)	0.016 (0.006; 0.025)	0.015 (0.008; 0.027)	0.008 (0.004; 0.019)	0.008 (0.004; 0.017)
AI	6.08 (3.49; 10.76)	7.41 (2.89; 10.84)	6.76 (3.73; 13.03)	3.88 (1.65; 10.39)	3.4 (1.6; 8.17)
VD DCP					
δ_abs_	0.007 (0.003; 0.013)	0.012 (0.006; 0.018)	0.009 (0.006; 0.016)	0.007 (0.002; 0.015)	0.014 (0.006; 0.016)
AI	2.91 (1.3; 5.3)	4.94 (2.4; 7.61)	3.64 (2.45; 7.09)	3.35 (0.86; 6.36)	6.26 (2.85; 8.02)
VD SCP IR					
δ_abs_	0.027 (0.01; 0.041)	0.027 (0.014; 0.041)	0.033 (0.021; 0.046)	0.022 (0.007; 0.032)	0.018 (0.012; 0.03)
AI	12.21 (4.2; 19.91)	12.69 (6.28; 19.34)	16.47 (10.66; 22.44)	10.58 (3.95; 17.79)	8.12 (5.92; 16.88)
VD SCP OR					
δ_abs_	0.015 (0.008; 0.023)	0.013 (0.009; 0.025)	0.012 (0.006; 0.027)	0.012 (0.008; 0.026)	0.016 (0.008; 0.02)
AI	4.8 (2.78; 7.62)	4.43 (3.04; 8.46)	4.09 (2.08; 8.81)	3.94 (2.95; 8.73)	4.95 (2.67; 6.65)
VD DCP IR					
δ_abs_	0.021 (0.009; 0.032)	0.032 (0.008; 0.051)	0.022 (0.011; 0.044)	0.022 (0.017; 0.05)	0.013 (0.004; 0.028)
AI	8.99 (3.09; 12.4)	11.32 (2.95; 20.87)	8.85 (4.38; 15.63)	8.11 (6.25; 19.58)	4.81 (1.57; 10.44)
VD DCP OR					
δ_abs_	0.008 (0.005; 0.014)	0.012 (0.009; 0.017)	0.01 (0.004; 0.017)	0.01 (0.006; 0.014)	0.011 (0.005; 0.014)
AI	3.09 (1.73; 4.93)	4.19 (3.15; 6.23)	3.53 (1.45; 6.27)	4.04 (2.06; 6.13)	3.92 (2.02; 5.36)
FD SCP					
δ_abs_	0.011 (0.004; 0.018)	0.008 (0.005; 0.017)	0.01 (0.005; 0.019)	0.007 (0.002; 0.021)	0.004 (0.002; 0.014)
AI	0.62 (0.23; 1.02)	0.45 (0.28; 0.95)	0.56 (0.26; 1.06)	0.4 (0.08; 1.2)	0.23 (0.11; 0.8)
FD DCP					
δ_abs_	0.005 (0.002; 0.01)	0.01 (0.007; 0.013)	0.005 (0.003; 0.011)	0.006 (0.001; 0.009)	0.007 (0.003; 0.012)
AI	0.28 (0.11; 0.56)	0.56 (0.39; 0.73)	0.25 (0.17; 0.63)	0.34 (0.06; 0.52)	0.37 (0.16; 0.68)
FAZA					
δ_abs_	0.021 (0.01; 0.032)	0.034 (0.016; 0.047)	0.041 (0.02; 0.057)	0.0355 (0.023; 0.06)	0.0765 (0.046; 0.133)
AI	9.38 (5.43; 16.15)	11.3 (7.66; 23.02)	17.25 (6.25; 30.3)	11.19 (9.38; 13.34)	22.82 (11.62; 48.28)
FAZP					
δ_abs_	0.107 (0.052; 0.187)	0.173 (0.06; 0.311)	0.137 (0.067; 0.306)	0.18 (0.089; 0.28)	0.478 (0.216; 0.887)
AI	6.29 (2.82; 10.08)	7.63 (3.46; 13.42)	7.5 (3.02; 17.86)	6.19 (4.45; 12.45)	19.61 (9.76; 27.46)
FAZC					
δ_abs_	0.049 (0.015; 0.08)	0.047 (0.022; 0.09)	0.073 (0.045; 0.114)	0.043 (0.023; 0.071)	0.096 (0.045; 0.17)
AI	6.17 (1.88; 10.95)	6.33 (2.74; 11.32)	10.58 (6.52; 15.1)	5.74 (3.17; 10.58)	15.37 (9.01; 26.96)

AI, asymmetry index.

Significant differences of asymmetry index between DR stages were revealed for FAZA (*P* = 0.005), FAZP (*P* = 0.0008), and FAZC (*P* = 0.0002). By comparing each DR stage with no DR, we found significantly higher asymmetry indices in eyes with moderate (*P* = 0.0154) and proliferative DR (*P* < 0.0001) compared to eyes with no DR for FAZA (*P* = 0.0154 and *P* < 0.0001, respectively) and FAZC (*P* = 0.0013 and *P* < 0.0001, respectively). Asymmetry index for FAZP was significantly higher in eyes with PDR compared to eyes without DR (*P* < 0.0001).

Analysis of δ_abs_ also revealed significant differences between DR stages and FAZA (*P* < 0.0001), FAZP (*P* < 0.0001), and FAZC (*P* = 0.0013). FAZA δ_abs_ was larger in all stages of DR compared to eyes with no DR (mild vs. no DR: *P* = 0.0445, moderate vs. no DR: *P* = 0.0006, severe vs. no DR: 0.0103, PDR vs. no DR: *P* < 0.0001). FAZP δ_abs_ was higher in severe and PDR compared to no DR (*P* = 0.0443 and *P* < 0.0001, respectively). FAZ circularity δ_abs_ was higher in moderate and PDR compared to no DR (*P* = 0.0011 and *P* = 0.0026, respectively).

### Right Versus Left Eye

**Figure 3.**
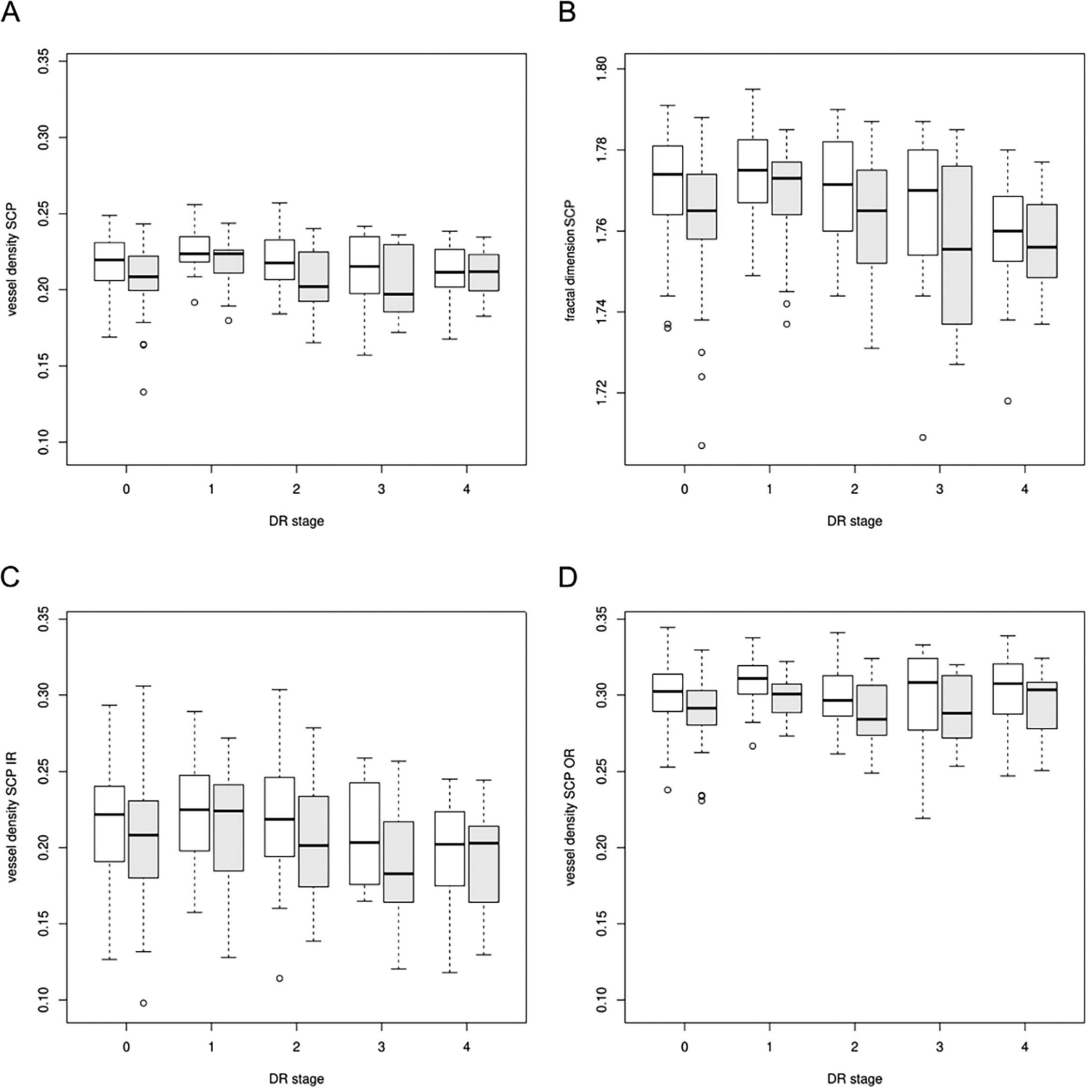
Boxplots of VD and FD in the SCP in different stages of DR (0 = no DR, 1 = mild DR, 2 = moderate DR, 3 = severe DR, 4 = proliferative DR) of right (*white*) and left eyes (*gray*). Vessel Density (**A**) and fractal dimension (**B**) are shown in the total area of the ETDRS grid. Furthermore, VD is specified in the IR (**C**) and OR (**D**).

**Figure 4.**
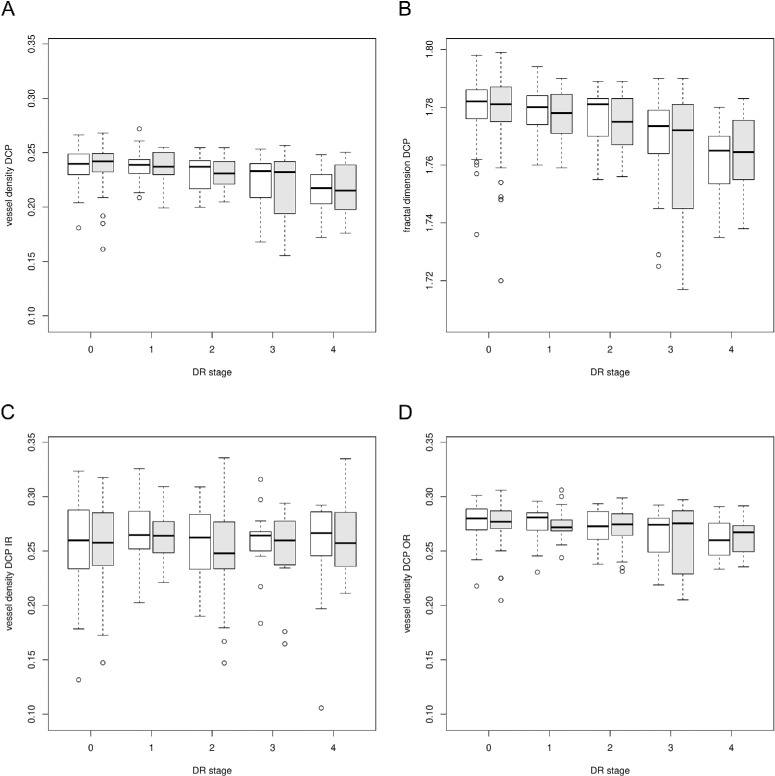
Boxplots of VD and FD in the DCP in different stages of DR (0 = no DR, 1 = mild DR, 2 = moderate DR, 3 = severe DR, 4 = proliferative DR) of right (*white*) and left eyes (*gray*). VD (**A**) and FD (**B**) are shown in the total area of the ETDRS grid. Furthermore, VD is specified in the IR (**C**) and OR (**D**).

The mixed model with VD in the SCP as dependent variable revealed significantly lower mean values on the left eye compared to the right eye (estimate [95% CI]: −0.009 [−0.01; −0.006], *P* < 0.0001). Similar results were found for the dependent variables SCP IR (estimate = −0.01 [−0.015; −0.004], *P* < 0.0001), SCP OR (estimate = −0.01 [−0.013; −0.007], *P* < 0.0001), as well as for FD in the SCP (estimate = −0.007 [−0.009; −0.005], *P* < 0.0001). This is visualized with boxplots in Figure 3. In the deep plexus, no statistically significant differences between left and right eye were found (VD in the DCP: estimate = 0.0002 [−0.0021; 0.0024], *P* = 0.9, DCP IR: −0.0026 [−0.008; 0.0028], *P* = 0.3, DCP OR: −0.0007 [−0.003; 0.0015], *P* = 0.5, FD in the DCP: −0.001 [−0.0025; 0.0006], *P* = 0.2) (shown in Fig. 4). No significant differences in FAZA between the right and left eye were found (*P* = 0.087).

The mixed model revealed a significant interaction between stage and side for FAZP (*P* = 0.0058) and FAZC (*P* = 0.025); thus comparisons between right and left eye were conducted for each stage separately. A significantly higher FAZP was found in eyes with PDR compared to patients without DR (estimate = −0.289 [0.155; 0.419], *P* < 0.0001). FAZC was lower in the left compared to the right eye in all stages of DR (as seen in Table 1). This difference was statistically significant in no DR (estimate = −0.03 [−0.046; −0.014, *P* < 0.0001), moderate DR (estimate = −0.049 [−0.079; −0.019], *P* = 0.0013), and PDR (estimate = −0.092 [−0.126; −0.057], *P* < 0.0001). No statistically significance was reached in mild (estimate = −0.034 [−0.069; 0.001], *P* = 0.0548) and severe DR (estimate = −0.023 [−0.064; 0.017], *P* = 0.2561). DR Stage, age, sex, and HbA1c were included as additional independent variables in the models.

### Association of Microvascular Parameters With DR Stage

Derived from the mixed models described above (comparisons of eyes), we found a significant difference of VD in the DCP between DR stages (*F* test, *P* < 0.0001). Severe versus no DR (estimate = −0.020 [−0.030; −0.011], *P* < 0.0001) and PDR versus no DR (estimate = −0.024 [−0.032; −0.016], *P* < 0.0001) showed significant reduction in VD. There was no significant difference between mild versus no DR (−0.003 [−0.011; 0.006], *P* = 0.5) and moderate versus no DR (estimate = −0.007 [−0.014; 0.0005], *P* = 0.07). Similar results were observed for the DCP OR (*F* test, *P* = 0.0005; severe vs. no DR: estimate = −0.015 [−0.024; −0.005], *P* = 0.003); PDR versus no DR (−0.015 [−0.024; −0.007], *P* < 0.0001) but not for the IR (*P* = 0.9). By descriptively analyzing in the localized VD in the DCP, the OR shows lower VD as DR stage progresses compared to the IR (as seen in Table 1). No significant VD difference between stages was found in the SCP (SCP: *P* = 0.2, SCP IR: *P* = 0.3, SCP OR: *P* = 0.5).

There was a significant difference of FD between stages in both the SCP (*F* test, *P* = 0.003) and DCP (*F* test, *P* < 0.0001). A significant reduction of FD was observed between severe versus no DR (SCP: estimate = −0.0074 [−0.015; −0.0002], *P* = 0.04; DCP: estimate = −0.015 [−0.021; −0.0085], *P* < 0.0001) and PDR versus no DR (SCP: estimate = −0.01 [−0.016; −0.0039], *P* = 0.002; DCP: estimate = −0.016 [−0.022; −0.011], *P* < 0.0001) in the SCP and DCP, respectively. FAZA was significantly larger in eyes with severe DR (estimate = 0.1159 [0.0274; 0.2044], *P* = 0.0106) and PDR (estimate = 0.1767 [0.0996; 0.2538], *P* < 0.0001) compared to eyes with no DR.

Because significant differences between stage and side were observed for FAZP and FAZC, stages were compared for both eyes separately. FAZP of both eyes was significantly higher in severe DR (right eye: estimate = 0.547 [0.184; 0.91], left eye: estimate = 0.56 [0.196; 0.923], both eyes: *P* = 0.003) and PDR (right eye: estimate = 0.772 [0.456; 1.089], left eye: estimate = 1.027 [0.711; 1.344], both eyes: *P* < 0.0001) compared to no DR.

In right eyes FAZC was significantly lower in severe DR (estimate = −0.06 [−0.109; −0.011], *P* = 0.0162) and PDR (estimate = −0.126 [−0.169; −0.084], *P* < 0.0001) compared to no DR. In left eyes FAZC was significantly lower in moderate (estimate = −0.045 [−0.083; −0.008], *P* = 0.0179), severe (estimate = −0.053 [−0.102; −0.004], *P* = 0.033), and PDR (estimate = −0.187 [−0.23; −0.145], *P* < 0.0001) compared to no DR.

## Discussion

The availability of retinal OCTA metrics provides additional markers for DR assessment and allows precise investigation of microvascular changes in systemic diseases such as DR. Because of the differences of hemodynamic stress between right and left carotid arteries we hypothesized that this finding extends to retinal blood flow parameters in diabetic patients. We found an independent intereye difference of VD and FD in the SCP, with the left eye showing reduced values compared to the right eye. Furthermore, FAZC was more affected in left compared to right eyes.

Literature is scarce on intereye differences in patients with DR. Although diabetes is a systemic disease, eyes can be affected differently and time delayed. It has been shown that patients with DR detected on CF of both eyes had a higher risk of disease progression than patients with DR in one eye only.^17^ Zhao et al.^18^ used an asymmetry index to evaluate differences between both eyes of 258 patients with DR using 3 × 3 mm OCTA macular scans. They found that asymmetry in the SCP and DCP was significantly larger in PDR compared to patients with no diabetes, diabetic patients without and with NPDR. However, they did not divide NPDR into subgroups. By evaluating the asymmetry index in different stages of DR in our cohort, we detected no significant difference between advanced and early stages. However, by comparing different macular areas, we found that asymmetry between eyes was higher in the IR of the ETDRS grid compared to the OR. This highlights potential differences regarding the retinal area evaluated as we analyzed a larger area of 6 × 6 mm in contrast to previously reported results of 3 × 3 mm with the central macula potentially being more affected by intereye divergence. In line with Zhao et al.^18^ we found higher asymmetries of the evaluated FAZ parameters between both eyes in more advanced DR stages highlighting that FAZ size as well as circularity change at a different rate.

By analyzing absolute differences between eyes, we found a significantly lower FD and VD in the SCP of the left compared to the right eye. This difference was independent of DR stage and might be due to anatomic differences. FAZC was lower in left compared to right eyes without DR, moderate DR, and PDR. Although descriptively left eyes were less circular in all groups (as seen in Table 1), this difference failed to reach significance in patients with mild and severe DR which could be explained by the smaller group sizes (19 and 14 patients, respectively). Higher FAZPs were observed in the left compared the right eye in patients with PDR.

A recent study evaluated changes to the right and left carotid artery including lumen diameter, intimal-media thickness, and other risk markers for atherosclerosis or cerebrovascular disease in 250 patients with diabetes and hypertension. They found significant differences of the left common carotid artery versus the right such as an increase of lumen diameter and intimal-media thickness, as well as higher pulsatility and resistance indexes of the left internal carotid artery in younger patients. They concluded that the anatomic origin of the left common carotid artery, which originates directly from the aortic arch, may cause more stress to the left internal carotid artery.^19^ An earlier study evaluated 102 untreated hypertensive patients and also found a higher intima-media thickness and flow velocity in the left common carotid artery compared to the right as well as a higher incidence of cerebrovascular stroke at the left side. They also suggest that this is related to hemodynamic stress and intimal damage of the left carotid artery.^30^ Because the ophthalmic artery is the first branch of the internal carotid artery, changes to this vessel can influence its branches and, hence, their supplying tissues.

To our knowledge, this is the first study to report this intereye difference in diabetic patients. Other studies have evaluated flow metrics of both eyes and found slower blood flow velocities in left eyes compared to right. However, this difference was descriptive without reaching significance.^31^^,^^32^ Because SCP is supplied by the central retinal artery directly whereas deeper layers are supplied by vertical anastomoses from the SCP, anatomic hemodynamic differences as described above could affect the SCP to a greater extent.^33^^,^^34^

Various authors have assessed VD and FD of the different capillary plexuses irrespective of intraindividual differences in DR with absolute results being hard to compare due to different imaging modalities, field of views, and calculation methods. It has been reported that lower VD and FD were associated with more advanced DR stages or disease progression.^6^^,^^8^^,^^9^ Dupas et al.^10^ described that capillary loss in patients with DR was more pronounced in the DCP. Sun et al.^9^ reported that lower VD in the SCP was associated with DME development and in the DCP with DR progression. By analyzing all eyes, we also found a reduction of the DCP in advanced compared to early DR stages, which was not present in the SCP. However, the here-reported significant intereye difference in the SCP may be taken into account in future microvascular blood-flow analyses of the microvasculature of patients with DR.

FAZ parameters were frequently analyzed in patients with DR, describing larger FAZ sizes and more irregular shapes as disease progresses.^9^^,^^13^^,^^16^ Our results support these findings as patients in more advanced stages showed a larger FAZA, higher FAZP, and lower FAZC. However, measurements of size such as area and perimeter were previously discussed to be less accurate due to a high variability than parameters of FAZ shape such as circularity.^14^ In our analysis, FAZC of the left eye was significantly lower in moderate, severe, and proliferative DR compared to no DR, whereas in right eyes these differences were only noticeable in severe and PDR compared to no DR. This finding could indicate an earlier impairment of the FAZ microvasculature of the left eye.

Limitations of this study include its cross-sectional character and the exploratory analysis of the data acquired. Additionally, advanced DR stages show higher rate of motion artifacts and segmentation errors which might influence vascular metrics.^35^ However, we performed image quality assessment by specialists to include only images of sufficient quality. Although we included only patients with myopia or hyperopia of less than 3 diopters, axial eye length was not measured potentially causing differences in FAZA and FAZP measurements between eyes. Therefore FAZC is more accurate because the ratio between both parameters corrects for size differences between measurements.

Although we found a significant reduction of the SCP, as well as a more irregular FAZ in the left compared to the right eye, we can only speculate about the cause. Further longitudinal studies are needed to evaluate anatomic influences on intereye differences which have already been shown to affect the carotid arteries.

OCTA provides important microvascular blood-flow metrics to assess ocular changes in systemic diseases such as DR. Our results reveal a significant intereye difference of the SCP and FAZC, which is markedly reduced in the left compared to the right eye of patients although presenting with the same clinical DR stage. This intereye difference might be caused by higher hemodynamic stress to the left carotid artery and needs to be investigated in future studies.

## References

[1] Teo ZL, Tham YC, Yu M, et al. Global prevalence of diabetic retinopathy and projection of burden through 2045: systematic review and meta-analysis. *Ophthalmology*. 2021; 128: 1580–1591. 33940045 10.1016/j.ophtha.2021.04.027

[2] Saeedi P, Petersohn I, Salpea P, et al. Global and regional diabetes prevalence estimates for 2019 and projections for 2030 and 2045: results from the International Diabetes Federation Diabetes Atlas, 9th edition. *Diabetes Res Clin Pract**.* 2019; 157: 107843. 31518657 10.1016/j.diabres.2019.107843

[3] Yau JWY, Rogers SL, Kawasaki R, et al. Global prevalence and major risk factors of diabetic retinopathy. *Diabetes Care*. 2012; 35: 556–564. 22301125 10.2337/dc11-1909 PMC3322721

[4] Jampol LM, Glassman AR, Sun J. Evaluation and care of patients with diabetic retinopathy. *N Engl J Med*. 2020; 382: 1629–1637. 32320570 10.1056/NEJMra1909637

[5] Early Treatment Diabetic Retinopathy Study Research Group. Grading diabetic retinopathy from stereoscopic color fundus photographs—an extension of the modified Airlie House classification. ETDRS report number 10. *Ophthalmology*. 1991; 98: 786–806. 2062513

[6] Zeng Y, Cao D, Yu H, et al. Early retinal neurovascular impairment in patients with diabetes without clinically detectable retinopathy. *Br J Ophthalmol*. 2019; 103: 1747–1752. 30674454 10.1136/bjophthalmol-2018-313582

[7] Zahid S, Dolz-Marco R, Freund KB, et al. Fractal dimensional analysis of optical coherence tomography angiography in eyes with diabetic retinopathy. *Investig Ophthalmol Vis Sci*. 2016; 57: 4940–4947. 27654421 10.1167/iovs.16-19656

[8] Xie N, Tan Y, Liu S, et al. Macular vessel density in diabetes and diabetic retinopathy with swept-source optical coherence tomography angiography. *Graefes Arch Clin Exp Ophthalmol*. 2020; 258: 2671–2679. 32661699 10.1007/s00417-020-04832-3

[9] Sun Z, Tang F, Wong R, et al. OCT angiography metrics predict progression of diabetic retinopathy and development of diabetic macular edema: a prospective study. *Ophthalmology*. 2019; 126: 1675–1684. 31358386 10.1016/j.ophtha.2019.06.016

[10] Dupas B, Minvielle W, Bonnin S, et al. Association between vessel density and visual acuity in patients with diabetic retinopathy and poorly controlled type 1 diabetes. *JAMA Ophthalmol**.* 2018; 136: 721–728. 29800967 10.1001/jamaophthalmol.2018.1319 PMC6136049

[11] Mokrane A, Zureik A, Bonnin S, et al. Retinal sensitivity correlates with the superficial vessel density and inner layer thickness in diabetic retinopathy. *Invest Ophthalmol Vis Sci*. 2021; 62: 28–28. 10.1167/iovs.62.14.28 PMC8648065 34846517

[12] Balaratnasingam C, Inoue M, Ahn S, et al. Visual acuity is correlated with the area of the foveal avascular zone in diabetic retinopathy and retinal vein occlusion. *Ophthalmology*. 2016; 123: 2352–2367. 27523615 10.1016/j.ophtha.2016.07.008

[13] Mansour AM, Schachat A, Bodiford G, Haymond R. Foveal avascular zone in diabetes mellitus. *Retina*. 1993; 13: 125–128. 8337493 10.1097/00006982-199313020-00006

[14] Shiihara H, Terasaki H, Sonoda S, et al. Objective evaluation of size and shape of superficial foveal avascular zone in normal subjects by optical coherence tomography angiography. *Sci Rep*. 2018; 8: 1–9. 29973663 10.1038/s41598-018-28530-7 PMC6031610

[15] Linderman RE, Muthiah MN, Omoba SB, et al. Variability of foveal avascular zone metrics derived from optical coherence tomography angiography images. *Transl Vis Sci Technol*. 2018; 7: 20. 10.1167/tvst.7.5.20 PMC6166903 30280005

[16] Krawitz BD, Mo S, Geyman LS, et al. Acircularity index and axis ratio of the foveal avascular zone in diabetic eyes and healthy controls measured by optical coherence tomography angiography. *Vision Res*. 2017; 139: 177–186. 28212983 10.1016/j.visres.2016.09.019

[17] Scanlon PH, Stratton IM, Histed M, Chave SJ, Aldington SJ. The influence of background diabetic retinopathy in the second eye on rates of progression of diabetic retinopathy between 2005 and 2010. *Acta Ophthalmol*. 2013; 91: 335–339. 23551550 10.1111/aos.12074 PMC3798105

[18] Zhao T, Laotaweerungsawat S, Chen Y, Liu X, Liu D, Stewart JM. Right versus left eye asymmetry of microvasculature in diabetes revealed by optical coherence tomography angiography. *Sci Rep*. 2023; 13: 1–7. 37291258 10.1038/s41598-023-36058-8 PMC10250307

[19] Al-Sabbagh AA, Essa SI, Saleh AZ. A comparative study of the right and left carotid arteries in relation to age for patients with diabetes and hypertension. *J Vasc Ultrasound*. 2022; 46: 118–121.

[20] Wilkinson CP, Ferris FL III, Klein RE, et al. Proposed international clinical diabetic retinopathy and diabetic macular edema disease severity scales. *Ophthalmology*. 2003; 110: 1677–1682. 13129861 10.1016/S0161-6420(03)00475-5

[21] Coscas F, Sellam A, Glacet-Bernard A, et al. Normative data for vascular density in superficial and deep capillary plexuses of healthy adults assessed by optical coherence tomography angiography. *Invest Ophthalmol Vis Sci*. 2016; 57: OCT211–OCT223. 27409475 10.1167/iovs.15-18793

[22] Yu J, Jiang C, Wang X, et al. Macular perfusion in healthy Chinese: an optical coherence tomography angiogram study. *Invest Ophthalmol Vis Sci*. 2015; 56: 3212–3217. 26024105 10.1167/iovs.14-16270 PMC4455309

[23] You Q, Freeman WR, Weinreb RN, et al. Reproducibility of vessel density measurement with optical coherence tomography angiography in eyes with and without retinopathy. *Retina*. 2017; 37: 1475–1482. 27930458 10.1097/IAE.0000000000001407 PMC5902313

[24] Stino H, de Llano Pato E, Steiner I, et al. Macular microvascular perfusion status in hypertensive patients with chronic kidney disease. *J Clin Med*. 2023; 12: 5493. 37685559 10.3390/jcm12175493 PMC10488526

[25] Yu S, Lakshminarayanan V. Fractal dimension and retinal pathology: a meta-analysis. *Appl Sci*. 2021; 11: 1–23.

[26] Fractalyse. Version 3.0-0.9.1. Available at: https://sourcesup.renater.fr/www/fractalyse/. Accessed August 31, 2023.

[27] Grieshop J, Gaffney M, Linderman RE, Cooper RF, Carroll J. The shape of the foveal avascular zone: when a circle isn't round. *Transl Vis Sci Technol*. 2023; 12: 3–5. 10.1167/tvst.12.6.26 PMC10309160 37378965

[28] Kim K, Kim ES, Yu SY. Optical coherence tomography angiography analysis of foveal microvascular changes and inner retinal layer thinning in patients with diabetes. *Br J Ophthalmol*. 2018; 102: 1226–1231. 29259019 10.1136/bjophthalmol-2017-311149

[29] Ristl R . mmmgee: Simultaneous Inference for Multiple Linear Contrasts in GEE Models. R package version 1.20. 2019. Available at: https://CRAN.R-project.org/package=mmmgee. Accessed September 26, 2023.

[30] Rodríguez Hernández SA, Kroon AA, Van Boxtel MP, et al. Is there a side predilection for cerebrovascular disease? *Hypertension*. 2003; 42: 56–60. 12810754 10.1161/01.HYP.0000077983.66161.6F

[31] Kimura I, Shinoda K, Tanino T, Ohtake Y, Mashima Y, Oguchi Y. Scanning laser Doppler flowmeter study of retinal blood flow in macular area of healthy volunteers. *Br J Ophthalmol*. 2003; 87: 1469–1473. 14660455 10.1136/bjo.87.12.1469 PMC1920574

[32] Landa G, Jangi AA, Garcia PMT, Rosen RB. Initial report of quantification of retinal blood flow velocity in normal human subjects using the Retinal Functional Imager (RFI). *Int Ophthalmol*. 2012; 32: 211–215. 22484724 10.1007/s10792-012-9547-z

[33] Campbell JP, Zhang M, Hwang TS, et al. Detailed vascular anatomy of the human retina by projection-resolved optical coherence tomography angiography. *Sci Rep*. 2017; 7: 1–11. 28186181 10.1038/srep42201 PMC5301488

[34] Provis JM . Development of the primate retinal vasculature. *Prog Retin Eye Res*. 2001; 20: 799–821. 11587918 10.1016/s1350-9462(01)00012-x

[35] Cui Y, Zhu Y, Wang JC, et al. Imaging artifacts and segmentation errors with wide-field swept-source optical coherence tomography angiography in diabetic retinopathy. *Transl Vis Sci Technol*. 2019; 8. 10.1167/tvst.8.6.18 PMC6859832 31772829

